# Supplementary material for: A Global Screen for Assembly State Changes of the Mitotic Proteome by SEC-SWATH-MS
Source: Cell Syst. 2020 Feb 26;10(2):133–155.e6. doi: 10.1016/j.cels.2020.01.001 (PMC7042714; doi:10.1016/j.cels.2020.01.001)

P10644 | KAP0_HUMAN | PRKAR1A PKR1 PRKAR1 TSE1
Monomer MW [kDa]: 42.982 Monomer expected elution fraction: 48

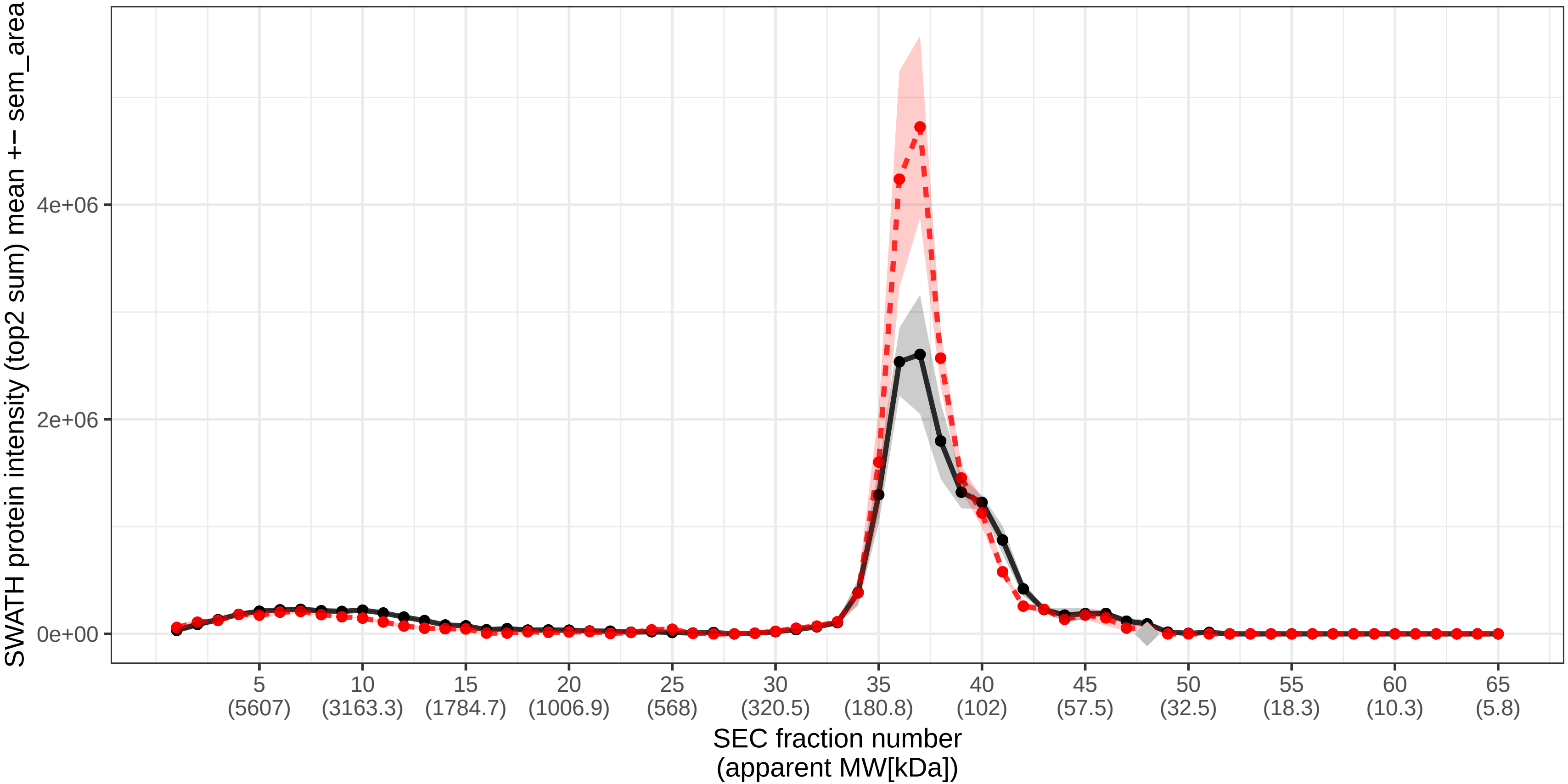

Supplement: Data S1. SEC-SWATH-MS Protein Chromatograms, Related to Figure 1 [file mmc6.zip › SECchrom_P10644_KAP0_HUMAN_PRKAR1A_PKR1_PRKAR1_TSE1.pdf]
